# Intelligent Residential Environments Catering to Dementia‐Friendly Needs

**DOI:** 10.1002/alz.085672

**Published:** 2025-01-09

**Authors:** Arshia A Khan

**Affiliations:** ^1^ University of Minnesota Duluth, Duluth, MN USA

## Abstract

This study was approved by the ethics review board at the University of Minnesota. In conclusion, the successful design and testing of an intelligent living space tailored for dementia care were conducted in a controlled lab environment with healthy participants. The primary aim was to assess the viability of integrating robots, wearable sensors, and spatial technology to support the well‐being of individuals affected by dementia. Utilizing pressure sensors in chairs alongside the robot’s emotion detection system effectively evaluated participants' moods, aligning with self‐reported moods in 95∖% of cases. Additionally, door sensors accurately detected exiting behavior 100∖% of the time, while wall‐mounted PIR sensors successfully identified wandering behavior. Wearable sensors, including EDA and blood pressure sensors, coupled with robot interactions, detected physiological changes during various activities, indicating the most significant impact during interactions with the robot. These findings suggest that an intelligent living space specifically designed for dementia care can effectively preserve the quality of life for affected individuals, particularly through robot interactions, showcasing promising effectiveness.

